# Dietary Protein Regulates Female Estrous Cyclicity Partially via Fibroblast Growth Factor 21

**DOI:** 10.3390/nu15133049

**Published:** 2023-07-06

**Authors:** Yaxue Cao, Min Yang, Jie Song, Xuemei Jiang, Shengyu Xu, Lianqiang Che, Zhengfeng Fang, Yan Lin, Chao Jin, Bin Feng, De Wu, Lun Hua, Yong Zhuo

**Affiliations:** 1 Key Laboratory for Animal Disease Resistant Nutrition of the Ministry of Education, Animal Nutrition Institute, Sichuan Agricultural University, Chengdu 611130, China; cyx9980@163.com (Y.C.); yang040101@yeah.net (M.Y.); 71310@sicau.edu.cn (X.J.); shengyuxu@sicau.edu.cn (S.X.); che.lianqiang@sicau.edu.cn (L.C.); zfang@sicau.edu.cn (Z.F.); linyan@sicau.edu.cn (Y.L.); jinchao@sicau.edu.cn (C.J.); fengbin@sicau.edu.cn (B.F.); wude@sicau.edu.cn (D.W.); 2 Pet Nutrition and Health Research Center, Chengdu Agricultural College, Chengdu 611130, China

**Keywords:** protein restriction, estrous cycle, FGF 21, *Kiss*-1

## Abstract

Fibroblast growth factor 21 (FGF21), a hormone predominantly released in the liver, has emerged as a critical endocrine signal of dietary protein intake, but its role in the control of estrous cyclicity by dietary protein remains uncertain. To investigated the role of FGF21 and hypothalamic changes in the regulation of estrous cyclicity by dietary protein intake, female adult Sprague-Dawley rats with normal estrous cycles were fed diets with protein contents of 4% (P4), 8% (P8), 13% (P13), 18% (P18), and 23% (P23). FGF21 liver-specific knockout or wild-type mice were fed P18 or P4 diets to examine the role of liver FGF21 in the control of estrous cyclicity. Dietary protein restriction resulted in no negative effects on estrous cyclicity or ovarian follicular development when the protein content was greater than 8%. Protein restriction at 4% resulted in decreased bodyweight, compromised *Kiss*-1 expression in the hypothalamus, disturbed estrous cyclicity, and inhibited uterine and ovarian follicular development. The disturbed estrous cyclicity in rats that received the P4 diet was reversed after feeding with the P18 diet. Liver *Fgf21* mRNA expressions and serum FGF21 levels were significantly increased as dietary protein content decreased, and loss of hepatic FGF21 delayed the onset of cyclicity disruption in rats fed with the P4 diet, possibly due to the regulation of insulin-like growth factor-1. Collectively, severe dietary protein restriction results in the cessation of estrous cyclicity and ovarian follicle development, and hepatic FGF21 and hypothalamic *Kiss*-1 were partially required for this process.

## 1. Introduction

With the rising incidence of obesity worldwide, greater emphasis has been placed on vegetarian diets [1,2,3], which consist of low-fat, low-protein, high-fiber, and vitamin-rich foods. A low protein intake has been associated with improved metabolic health [4,5,6,7], long lifespan [8,9], and even anti-cancer immunosurveillance [10]. Although women on vegetarian diets are healthier than those on omnivore diets in terms of the body mass index and other related parameters, they may face menstrual irregularities and ovulatory disturbances [11,12], probably due to the limited intake of dietary protein.

Nutrition and fertility are closely related, and reproduction occurs only in favorable conditions with respect to nutritional sufficiency. Decreased reproductive function in cases of nutritional deprivation is an adaptive process that is conserved and essential for the survival of species. The mechanisms that inhibit fertility during nutrient restriction are complex, and disordered reproductive functions are due to alternations in metabolic status and disruptions within the hypothalamic–pituitary–gonadal (HPG) axis [13,14]. However, the cellular and molecular mechanisms related to the effects of dietary protein intake on reproduction remain poorly understood. Estrous cyclicity is reflected in follicular changes under the action of gonadotropin-releasing hormone (GnRH) and preovulatory gonadotropin surges, which are induced by a positive feedback of estradiol (E_2_) released from matured ovarian follicles, a process essential for reproduction. Hypothalamic GnRH neurons are believed to be the main regulators of the HPG axis [15].

Recent studies indicate that kisspeptins, the peptide products of the *Kiss*-1 gene and their putative receptor G-protein-coupled receptor 54 (GPR54) constitute the gatekeeper function of GnRH, and both proteins are involved in pubertal onset, estrous cyclicity, and ovulation [16,17]. Kisspeptins trigger the preovulatory luteinizing hormone (LH) surge through direct action on GnRH neurons, while immunoneutralization of kisspeptin abolishes the proestrus LH surge and disrupts estrous cyclicity [18,19]. *Kiss*-1-expressing neurons may represent the connection between systemic metabolic signals and reproductive function, because metabolic hormones, such as leptin [20], fibroblast growth factor 21 (FGF21) [21], and insulin-like growth factor-1 (IGF-1) [22], can directly influence hypothalamic *Kiss*-1 gene expression and HPG axis activity. Importantly, FGF21 is a hormone predominantly released in the liver that has emerged as a critical endocrine signal mediating the effect of dietary protein intake on ovarian primordial follicle activation [23]. However, whether these metabolic hormones are involved in the effect of dietary protein intake on the HPG axis remains unknown.

Therefore, we hypothesized that the level of dietary protein intake can induce changes in metabolic status, thereby affecting estrous cyclicity and fertility. To test this, female adult Sprague-Dawley rats were fed diets with five different levels of protein content ranging from 4% to 23%. Then, FGF21 liver-specific knockout or wild-type mice were used to examine the role of liver FGF21 in the control of estrous cyclicity by dietary protein intake. Moreover, we examined whether the reproductive dysfunction induced by a dietary protein deficit can be reversed with a normal diet without negative effects on fertility.

## 2. Materials and Methods

### 2.1. Study Approval

All experimental procedures were performed in accordance with the National Research Council’s Guide for the Care and Use of Laboratory Animals. The Institutional Animal Care and Research Committee of Sichuan Agricultural University approved this study (protocol number: SICAU-2015-035 for rat study and SICAU-2021-011 for mouse study).

### 2.2. Animals and Diets

Eleven-week-old female Sprague-Dawley rats were purchased from Chengdu Dossy Experimental Animal Co., Ltd. (Chengdu, China) and housed individually in cages at 20–22 °C with 12:12 h light:dark cycles for 1 week to allow the animals to adapt to laboratory conditions. The generation of FGF21 liver-specific knockout (FGF21LKO) mice has been described previously [23,24,25]. Briefly, FGF21^loxp/loxp^ mice (022361; The Jackson Laboratory, Bar Harbor, ME, USA) were mated with Alb-Cre mice (J003574; Model Animal Research Center, Nanjing University, Nanjing, China) to generate FGF21^Liver+/−,Alb-Cre^ mice. FGF21^Liver−/−,Alb-Cre^ mice were generated by crossing FGF21^Liver+/−,Alb-Cre^ mice with FGF21^loxp/loxp^ mice. Littermates of FGF21^loxp/loxp^ mice were used as controls. The efficiency of hepatic FGF21 knockout has been demonstrated previously [23,24,25].

Diets for rats were formulated to contain 18% protein (P18, *n* = 18) following AIN-93 standards with minor modifications; three protein-restricted diets at 4% (P4, *n* = 18), 8% (P8, *n* = 18), and 13% (P13, *n* = 18); and one protein-excess diet at 23% (P23, *n* = 18) as previously described with minor modifications [23]. Casein was the sole protein source in the present study, and the level of protein content was increased by graded inclusion of casein. Food intake was measured daily, and bodyweight was recorded once a week. Estrous cycles were checked by performing daily vaginal smears as previously described [26]. The full estrous cycle occurs over 4 or 5 days and can be divided into four stages: proestrus (P), estrus (E), metestrus (M), and diestrus (D). Eventually, all treatment groups consisted of both cyclicity-normal rats and cyclicity-disturbed rats. P4 rats with cycle irregularity were sacrificed when the percentage of cyclicity-disturbed rats remained unchanged for at least 8 days. Meanwhile, rats in the other groups were sacrificed for sample collection. For the FGF21LKO mice study, 11-week-old wild-type (WT) or FGF21LKO mice were divided to receive the P18 diet or the P4 diet, and the cyclicity was then checked. The P4 and P18 diets were the same as those for rats. The P4 mice were sacrificed at the diestrus stage for sample collection when they showed a disrupted estrous cycle for at least 10 days.

### 2.3. Serum Amino Acid and Hormone Analyses

Blood samples were centrifuged for 10 min at 4000 rpm and 4 °C, and serum was collected and stored at −20 °C until analysis. Serum concentrations of FGF21, FSH, LH, E_2_, IGF-1, and leptin were estimated using specific enzyme-linked immunosorbent assay (ELISA) kits purchased from R&D Systems (Minneapolis, MN, USA). Serum concentrations of amino acids were determined using an automatic amino acid analyzer (Hitachi, Tokyo, Japan) as described in [23].

### 2.4. Tissue Collection

Brains were harvested immediately after sacrifice, and excess tissues were removed. Hypothalamus samples were collected as described previously [27]. Briefly, the hypothalami of rats were dissected by a horizontal incision about 2 mm in depth with the following limits: 1 mm anteriorly from the optic chiasm, the posterior border of the mamillary bodies, and the hypothalamic fissures. The uterus, ovaries, liver, and fat pads (subcutaneous inguinal, perigonadal, and dorsal abdominal fat pads around the kidneys) were removed and pruned for excess intestinal and other visceral tissues. The hypothalami, ovaries, liver, and fat pads from WT or FGF21LKO mice were collected as described [24]. All tissues were washed with pre-cooled phosphate-buffered saline three times, blotted with paper towels, and weighed.

### 2.5. Classification of Ovarian Follicles

Follicle types in ovarian cross sections were defined using previously established criteria as described previously [23]: primordial follicle, an oocyte with one layer of 3–6 flattened pre-granulosa cells; growing follicle, an oocyte surrounded by one or more layers of granulosa cells with no antrum formation; antral follicle, a large oocyte surrounded by multiple layers of granulosa cells with the presence of an antrum; atretic follicle, a degenerating oocyte or the presence of at least one pycnotic granulosa cell; and corpus luteum, a mature structure with no evidence of regressive changes. The primordial, primary, and early secondary follicles with visible nuclei were counted, and those without nuclei were excluded. However, late secondary follicles and antral follicles with diameters <1 mm, which were not easy to find in ovarian sections, were counted even if they did not have a visible nucleus [28]. The total numbers of all follicles were determined using three randomly selected center-tissue sections from each ovary and averaged.

### 2.6. Re-Feeding Experiment

To examine whether the adverse effects of protein restriction on reproductive function could be reversed, P4 rats with disturbed cyclicity were fed the P18 diet (P4 + P18) or the P4 diet (P4 + P4). Age-paired P18 rats (P18 + P18) were used as controls. Rats (*n* = 5–6 per group) in the P4 + P18 group were sacrificed for sample collection after the restoration of the estrous cycle. The fat pads (subcutaneous inguinal, perigonadal, and dorsal abdominal fat pads around the kidneys) were removed and weighed. The ovaries were paraffin-embedded, sectioned at 5 μm thickness, and stained with hematoxylin–eosin. Next, the female rats in P4 + P4 (*n* = 14), P4 + P18 (*n* = 15), and P18 + P18 (*n* = 15) groups were allowed to mate with fertile four-month-old male rats as described in [29]. Briefly, male rats were housed with female rats at a ratio of 1:1 from 21:00 to 07:00, and the day of vaginal plug was recorded as 0.5 day of pregnancy. The mating trial lasted for 14 days during which the dietary treatments continued. 

### 2.7. Real-Time Polymerase Chain Reaction Analysis

Total RNA was extracted from tissues using RNAiso Plus reagent (Takara, Dalian, China) according to the manufacturer’s instructions. The quality of total RNA was determined by the ratio of the absorbances at 260 and 280 nm (A260/280) and the visualization of 5sRNA, 18sRNA, and 28sRNA using agarose gel electrophoresis. Total RNA (1 μg) was used as a template to synthesize cDNA in a 20 μL reaction volume. Amplification and relative Ct values were determined in an ABI PRISM 7900HT Fast Real-Time PCR system (Thermo Fisher Scientific, Waltham, MA, USA) using SYB Green Real-Time PCR reagent (Takara). The cycle threshold (2^−ΔΔCt^) method was used to calculate the relative gene expression. The primer sequences are shown in Supplemental Table S1.

### 2.8. Statistical Analysis

Data were expressed as means with standard error and analyzed by one-way analysis of variance (ANOVA). When applicable, multiple comparisons were performed by Duncan’s method using SPSS 17.0 (SPSS Inc., Chicago, IL, USA). An unpaired Student’s *t*-test was performed to ascertain statistical significance in the re-feeding experiment. A chi-square test was used to analyze differences in the percentage of rats with disturbed cyclicity and the number of rats becoming pregnant in the re-feeding experiment. For the experiments involving WT or FGF21 liver-specific knockout mice, the data were analyzed using two-way ANOVA or Student’s *t*-test for multiple comparisons to determine differences between each group. Significance was accepted at *p* < 0.05.

## 3. Results

### 3.1. Protein Restriction or Excess Affects Bodyweight and Adipose Tissue Retention in Rats

**Figure 1:**
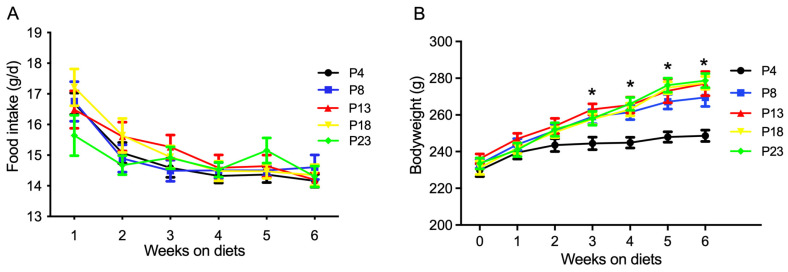
Food intake and bodyweight of rats fed varied amounts of protein. (**A**) Food intake of rats on diets for six weeks. (**B**) Bodyweight of rats on diets for six weeks. P4, P8, P13, P18, and P23 denote dietary protein contents at the levels of 4%, 8%, 13%, 18%, and 23%. Values are means ± S.E., *n* = 18 rats per group. * denotes that bodyweight in P4 rats was lower than the other groups (*p* < 0.05).

The effects of dietary protein level on the food intake and bodyweight of rats are presented in Figure 1. The food intake at different weeks was not affected by dietary protein level (Figure 1A). However, as shown in Figure 1B, the bodyweight of rats fed the P4 diet was significantly lower than that in the other groups at the end of the third, fourth, fifth, and sixth week (*p* < 0.05). The bodyweight of rats fed diets with a protein content greater than 8% was not affected (*p* > 0.05).

As shown in Table 1, the absolute and relative tissue weights of the uterus and ovaries were dose-dependently reduced for rats fed decreasing amounts of protein (*p* < 0.05). On the contrary, the weight of fat pads was greater for protein-restricted rats in P4, P8, and P13 groups compared with rats fed the P23 diet (*p* < 0.05). The absolute liver weight in PR4-group rats was significantly lower than that in the other groups (*p* < 0.05), but the liver weight percentage was not affected by the dietary protein content (*p* > 0.05).

### 3.2. Limited Protein Intake Disrupts Estrous Cyclicity and Follicular Development

**Figure 2:**
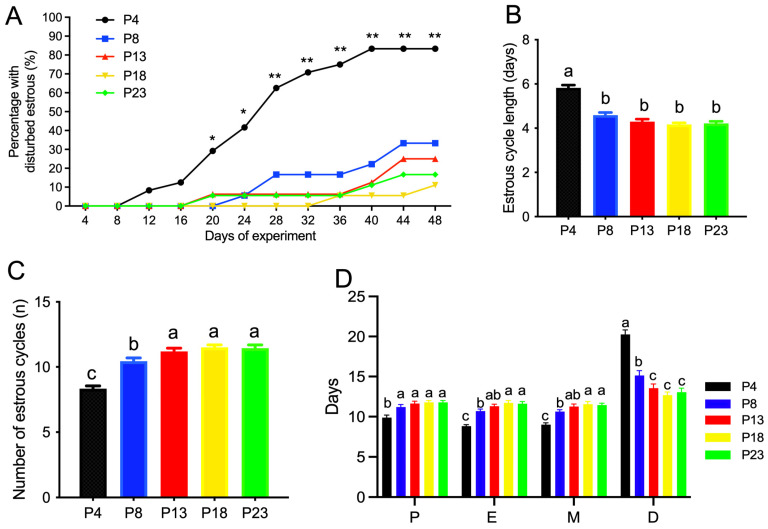
Estrous cyclicity of rats fed varied amounts of protein. (**A**), percentage of rats with disturbed estrous cyclicity. * denotes *p* < 0.05, and ** denotes *p* < 0.01, for P4 vs. P8 using Chi-square test; (**B**), average estrous cycle length. (**C**), number of estrous cycles within 48 days on diets. (**D**), average number of days rats stayed at each stage of estrous cyclicity. P, proestrus; E, estrus; M, metestrus; D, diestrus. Percentage of rats with disturbed estrous cyclicity at different days was analyzed by Chi-square test. P4, P8, P13, P18, and P23 denote dietary protein contents at the levels of 4%, 8%, 13%, 18%, and 23%. Values are means ± S.E., *n* = 18 rats per group. Volumes with different lowercase letters denote *p* < 0.05.

The estrous cyclicity in rats fed different amounts of protein is presented in Figure 2A–D and Supplemental Figure S1. As shown in Figure 2A and Supplemental Figure S1, rats fed the P4 diet showed a significantly increased percentage of cyclicity on day 20 of the experiment, and no difference was observed in the rats fed a diet with a protein content greater than 8%. The average estrous cycle length was significantly higher in P4-group rats compared with the other groups (Figure 2B), and an increased number of estrous cycles was observed in P4-group rats compared to P13-group rats within the 48-day experimental period (Figure 2C), which reached a plateau at 13% dietary protein. Rats fed protein-restricted diets, especially the P4 diet, showed fewer days at proestrus, estrus, or metestrus, but more days at diestrus (Figure 2D).

**Figure 3:**
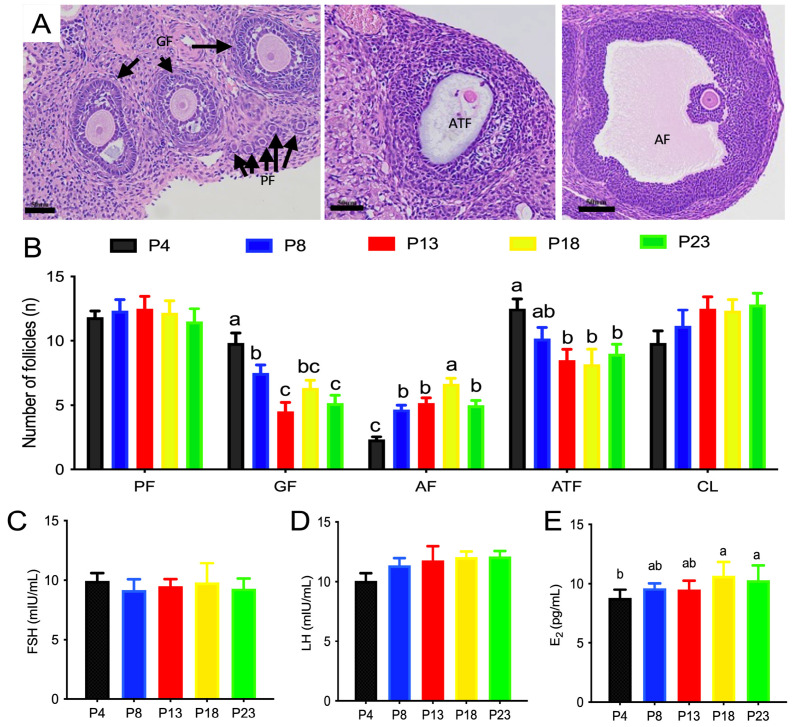
Number of ovarian follicles of rats fed varied amounts of protein. (**A**,**B**) are representative images of distribution of follicles at different developmental stages. PF denotes primordial follicles; GF denotes growing follicles; AF denotes antral follicles; ATF denotes atretic follicles; CL denotes corpus luteum. (**C**–**E**), serum levels of follicle-stimulating hormone (FSH), luteinizing hormone (LH), and estradiol (E_2_), respectively. Columns with different lowercase letters denote *p* < 0.05. P4, P8, P13, P18, and P23 denote dietary protein contents at the levels of 4%, 8%, 13%, 18%, and 23%, and *n* = 6 rats per group.

The classification of follicles of rats fed different amounts of protein is shown in Figure 3. The number of primordial follicles or corpus lutea was not affected by dietary protein content. P18-group rats had the highest number of antral follicles compared with the other groups (*p* < 0.05), whereas P4-group rats had the lowest number of antral follicles compared with the other groups (*p* < 0.05). The number of atretic follicles was greater in P4-group rats than that P13-, P18-, and P23-group rats.

### 3.3. Limited Protein Intake Disrupts Gene Expression in the HPG Axis

**Figure 4:**
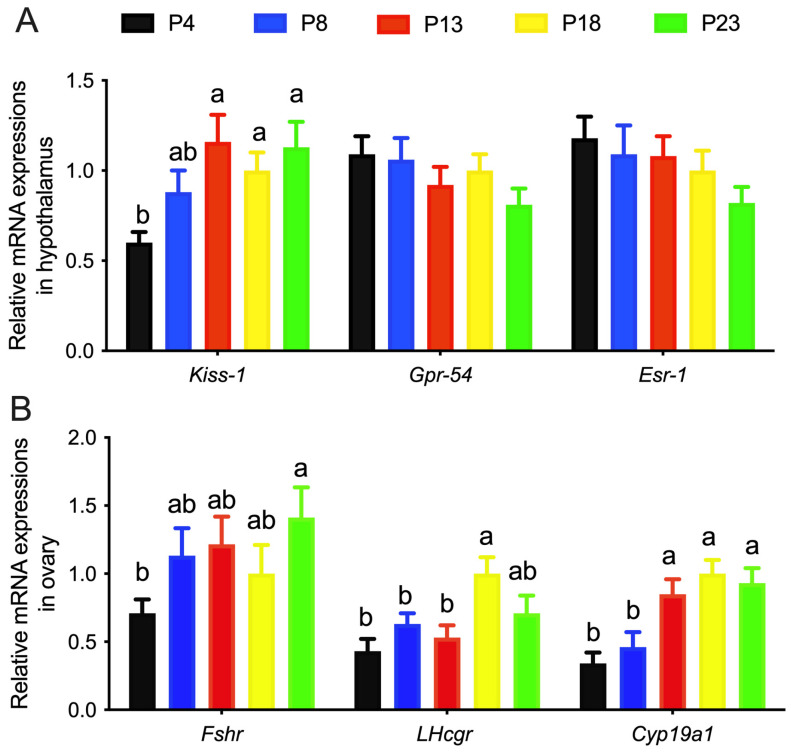
Relative mRNA expressions of genes in rats fed varied amounts of protein. (**A**), relative gene expressions in hypothalamus. (**B**), relative gene expressions in ovarian tissues. Columns with different lowercase letters denote *p* < 0.05. P4, P8, P13, P18, and P23 denote dietary protein contents at the levels of 4%, 8%, 13%, 18%, and 23%, and *n* = 6 rats per group.

The mRNA expression of *KiSS-1, Gpr-54*, and *Esr*-1 in the hypothalamus of rats is shown in Figure 4A. The mRNA expression of *Kiss*-1 in the hypothalamus in P4-group rats was significantly lower than that in the other groups, whereas the mRNA expression of *Gpr*-54 and *Esr*-1 was not affected. The mRNA expression of *Fshr, Lhcgr*, and *Cyp19a1* in the ovaries of rats was increased as the content of dietary protein increased (Figure 4B).

### 3.4. Protein Restriction-Induced Cyclicity Disruption Is Reversed by Feeding with a Normal Diet

**Figure 5:**
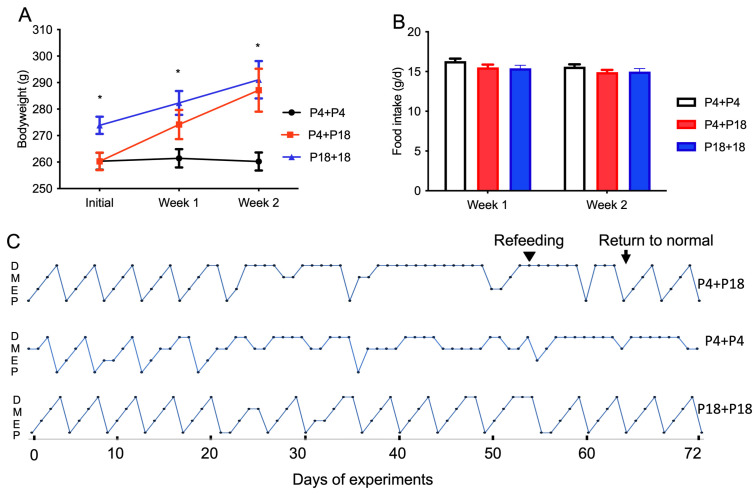
Estrous cyclicity of P4 rats fed normal amounts of protein in re-feeding trial. Bodyweight (**A**), food intake (**B**), and estrous cyclicity (**C**) in the re-feeding trial. P4 + P4, P4 rats continued the P4 diet during re-feeding phase; P4 + P18, P4 rats were re-fed with P18 diet; P18 + P18, P18 rats continued the P18 diet during re-feeding phase; P, proestrus; E, estrus; M, metestrus; D, diestrus. * denotes *p* < 0.05.

Initially, the bodyweight was lower in P4 + P4-group rats than that in P18-group rats (Figure 5A). However, after 2 weeks of feeding, the P4-group rats were fed the P18 diet, and they showed catch-up growth (Figure 5A) under similar food intake (Figure 5B). The P4-group rats on the P18 diet required about 11.17 days for estrous cycle recovery (Figure 5C). The return of normal estrous cyclicity in P4 + P18-group rats was accompanied by the recovery of ovarian, uterine, fat pad, and liver development (Supplemental Table S2).

**Figure 6:**
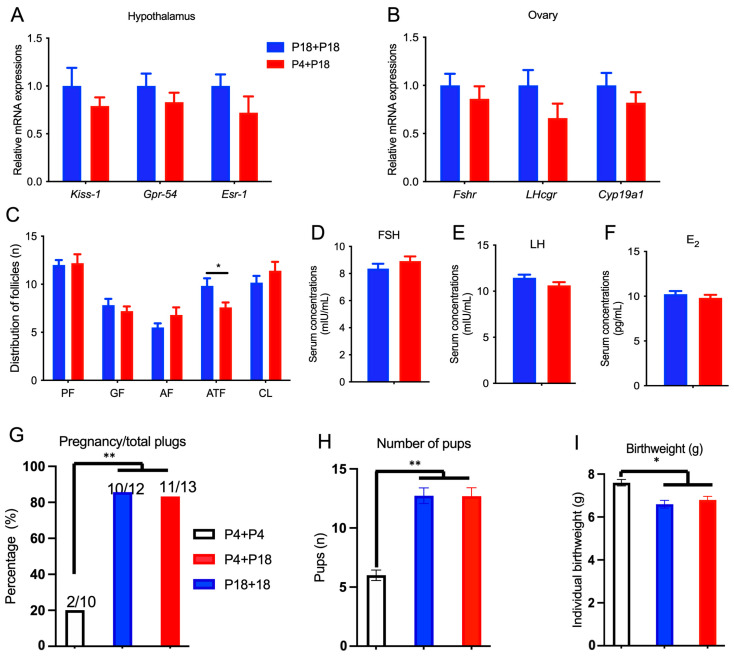
Re-feeding with normal protein diet rescued the fertility of rats. Relative gene expressions in hypothalamus (**A**), ovary (**B**), follicle development (**C**), and serum concentrations of FSH (**D**), LH (**E**), and E2 (**F**) in the re-feeding trial. The ratio of pregnancy to total plugs (**G**), number of pups (**H**), and birthweight (**I**) of the rats in the re-feeding trail. P4 + P4, P4 rats continued the P4 diet during re-feeding phase; P4 + P18, P4 rats were re-fed with P18 diet; P18 + P18, P18 rats continued the P18 diet during re-feeding phase; * denotes *p* < 0.05, and ** denotes *p* < 0.01.

The mRNA expression of *Kiss*-1, Gpr-54, and Esr-1 in the hypothalamus (Figure 6A) and the mRNA expression of Fshr, Lhcgr, and Cyp19a1 in the ovaries (Figure 6B) in P4 + P18-group rats were similar to those of P18 + P18-group rats after re-feeding. The number of primordial follicles, growing follicles, antral follicles, and corpus lutea were similar, but the number of atretic follicles in P4 + P18-group rats was lower than that in P18 + P18-group rats (Figure 6C). The secreted levels of FSH, LH, and E_2_ were similar (Figure 6D–F). Furthermore, a fertility test was conducted with rats of P4 + P18 and P18 + P18 groups. The percentage of pregnancies per plug and the number of pups was similar between P4 + P18 and P18 + P18 groups, but they were greater than those in P4 + P4 rats (Figure 6G,H). The birthweight of pups in the P4 + P4 group was greater than that in P4 + P18 and P18 + P18 groups (Figure 6I).

### 3.5. Limited Protein Intake Causes Significant Changes in Serum Amino Acid and Hormone Levels

Rats fed the P4 diet had lower serum concentrations of essential amino acids compared to the other groups, with the exception of methionine and isoleucine (Table 2). On the other hand, non-essential amino acid concentrations were elevated in rats when the protein content was less than 8%. The concentrations of asparagine, ornithine, histidine, and arginine were not affected by the dietary protein content. In general, as the dietary protein content increased from 4% to 23%, the ratio of total essential amino acids to total non-essential amino acids increased from 0.31 to 0.83. Furthermore, the restoration of estrous cyclicity and fertility for P4 + P18-group rats in the re-feeding experiment was associated with normal essential and non-essential amino acid concentrations in the serum compared to P18 + P18-group rats (Supplemental Table S3).

### 3.6. Liver FGF21 Is Partially Required for the Effect of Protein Restriction on Cyclicity

**Figure 7:**
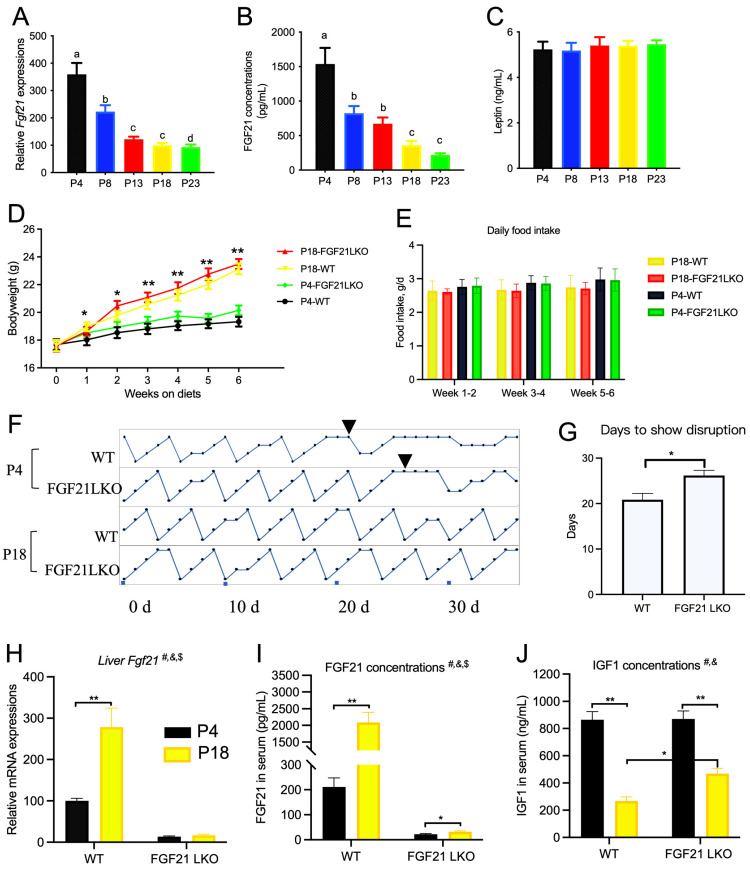
Role of liver FGF21 on the estrous cyclicity of mice fed the P4 diet. (**A**–**C**), liver *Fgf21* mRNA, serum FGF21, and leptin concentration for rats fed with different diets. Bodyweight (**D**) and food intake (**E**) of WT and FGF21LKO mice fed the P4 diet. Estrous cyclicity (**F**) and days to show estrous disruption (**G**). *Fgf21* mRNA in liver (**H**), FGF21 concentration in serum (**I**), and IGF1 concentration in serum (**J**). P4, P8, P13, P18, and P23 denote dietary protein contents at the levels of 4%, 8%, 13%, 18%, and 23%. ^#, &^ and ^$^ denote the significance of genotype, diet, and interaction of both. Columns with different lowercase letters denote *p* < 0.05. * denotes *p* < 0.05, and ** denotes *p* < 0.01.

Hepatic *Fgf21* expression and serum FGF21 concentration were significantly changed by changes in dietary protein content (Figure 7A,B). The serum leptin concentration was not affected (Figure 7C), despite differences in adipose tissue content between the groups. Therefore, 11-week-old WT and FGF21LKO mice were generated and fed the P4 or P18 diet. The bodyweight of mice fed the P18 diet was greater than that of mice fed the P4 diet (Figure 7D), and the average daily food intake was not affected (Figure 7E). Both WT and FGF21LKO mice fed the P18 diet had normal cyclicity (Figure 7F), whereas WT mice fed the P4 diet had disrupted estrous cyclicity at 20.8 days (Figure 7G,H). However, FGF21LKO mice fed the P4 diet required an additional 5.3 days for disrupted cyclicity compared to WT mice (Figure 7H). WT mice fed the P4 diet showed significantly higher *Fgf21* mRNA levels in the liver and higher FGF21 concentrations in serum (Figure 7I). Meanwhile, serum IGF1 concentration was decreased by the P4 diet, whereas serum IGF1 concentration was increased in FGF21LKO mice compared with WT mice fed the P4 diet (*p* < 0.05, Figure 7J).

**Figure 8:**
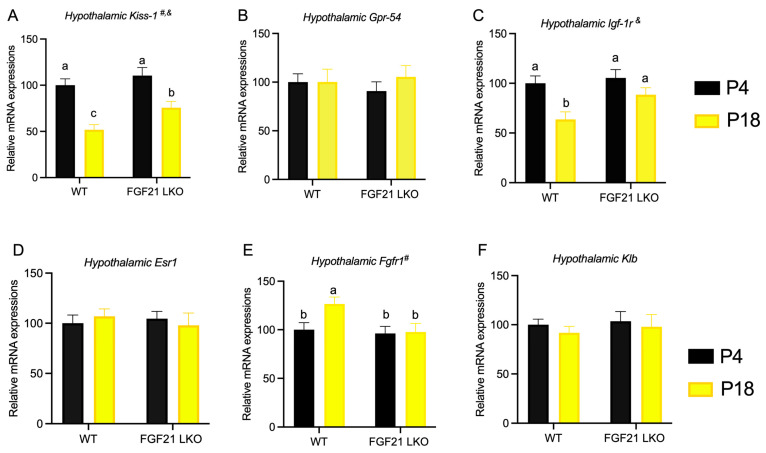
Role of liver FGF21 on the hypothalamic gene expressions of mice fed the P4 diet. Hypothalamic expressions of *Kiss-1*(**A**), *Gpr-54* (**B**), *Igf-1r* (**C**), *Esr-1* (**D**), *Fgfr1* (**E**), and *Klb* (**F**) were determined by RT-PCR. P4 and P18 denote dietary protein contents at the levels of 4% and 18%. ^#^ and ^&^ denote the significance of genotype and diet. Columns with different lowercase letters ^a,b^ denote *p* < 0.05.

As shown in Figure 8A–F, gene expression studies revealed that mice fed the P4 diet had decreased *Kiss-1* and *Igf-1r* expression in the hypothalamus compared with those fed the P18 diet. However, the loss of hepatic FGF21 expression resulted in elevated hypothalamic *Kiss-1* and *Igf-1r* expression in mice fed the P4 diet. The hypothalamic *Fgfr1* mRNA level in WT mice was increased by the P4 diet but was not altered by the P4 diet in FGF21LKO mice.

## 4. Discussion

Obesity is a serious health burden, especially for pregnant females. Upon pregnancy, obese women have a greater risk of developing gestational diabetes or pre-eclampsia than their lean counterparts [30]. Therefore, the Institute of Medicine in the United States recommends that obese women limit weight gain before or during pregnancy [31]. Consequently, an increasing focus has been placed on vegetarian diets in recent years, which predisposes vegetarians to have healthier body mass indices than omnivores. However, due to the limited selection of plant protein sources, this kind of dietary regimen can result in protein deficiency unless adequate precautions are taken [1,3] and can associate with menstrual irregularity and ovulatory disturbances [12,32,33]. Our results demonstrate that dietary protein restriction at 4% can disrupt estrous cyclicity, but this disruption can be reversed quickly by eating a normal diet.

The results of our study confirmed the hypothesis that protein restriction can induce a preference of nutrition partition toward adipose tissue despite similar energy intake. This is an adaptive response of mammals to adjust their needs for survival and reproduction. Notably, the inactivation of the HPG axis, characterized by the impaired development of ovarian and uterine tissues, was the leading cause of the cyclicity disturbances in this study. However, little is known about the molecular mechanisms mediating dietary protein restriction on the perturbation of the HPG axis. The metabolic status induced by different dietary regimens could be the main reason for the nutritional influence on HPG activity [13,14,20]. In the present study, rats fed the P4 diet had a significantly lower bodyweight after 3 weeks of feeding despite similar food intake, which may be due to a decreased body protein deposition rate and an increased tissue mobilization rate [6,7].

In the present study, one primary objective was to validate the dose-dependent effects of dietary protein content on the estrous cyclicity and ovarian follicular development.

The protein content of the P4 diet was below the need for peak efficiency of carcass protein gain (10% casein) [34]. Thus, the endogenous protein breakdown was increased to synthesize more critical proteins. In accordance with this, the protein-restricted rats showed elevated concentrations of serum non-essential amino acids as well as decreased essential amino acid concentrations as a result of protein degradation [35,36], which negatively affected body growth and protein synthesis. Notably, fat disposition was increased when dietary protein was limited despite lower bodyweight, indicating that mammals can adjust their nutrient partition to favor survival rather than reproduction under protein restriction [37,38]. Our results imply that alternations in nutrient partition with a preference for body fat deposition rather than reproduction is the main reason for the disturbed estrous cyclicity under protein restriction.

The perturbation of estrous cyclicity under protein restriction was closely associated with molecular and endocrinological changes at the level of the hypothalamus, pituitary gland, or gonads. Our previous study found that nutrient restriction delayed the age of puberty, which may be the result of compromised gene expression in the HPG axis [39]. In our study, decreased LH and E_2_ secretion was observed in rats fed the P4 diet, indicating that the compromised activity of the HPG axis may be the main reason for disturbed reproductive function. The *Kiss*-1–GPR54 complex, a newly discovered positive regulator of GnRH neurons, has been reported to control pubertal onset and HPG axis activation [16,17]. In the present study, hypothalamic *Kiss*-1 expression was compromised in rats fed a protein-restricted diet, which may have altered ovarian development given that the HPG axis is usually involved in this process. Furthermore, ovarian and uterine development was impaired in rats fed a protein-restricted diet. The important role of *Kiss*-1 in fertility was also evident in the re-feeding experiment. After rats were re-fed a normal diet, the P4-group rats with disturbed estrous cyclicity showed a return to normal cyclicity, which was associated with reversed hypothalamic *Kiss*-1 expression similar to the rats fed the P18 diet. Meanwhile, ovarian and uterine development was restored, and when the P4 rats were re-fed a normal diet, normal estrous cyclicity was associated with normal fertility. The ratio of pregnancy to total plugs, which is an indicator of the ability of rodents to become pregnant [29], was very low (20%) for P4 + P4 rats, but it was normal when P4-group rats were fed the P18 diet. However, the birthweight of offspring in the P4 + P4 group was greater than that in the other two groups, which was possibly due to the small litter size [40]. Nevertheless, our findings reveal that protein restriction induces a disruption on estrous cyclicity, which can be rapidly reversed without negative effects on the offspring.

Our results confirmed that protein restriction can induce atrophy of reproductive tissues and disruption of estrous cyclicity, and hypothalamic *Kiss*-1 gene expression plays a critical role in this process. However, it is unclear how kisspeptin is linked to metabolic status induced by nutritional restriction. In recent decades, FGF21 has emerged as a critical endocrine signaling molecule that mediates the effects of protein restriction on metabolic homeostasis [5,6,7,41]. The P4 diet was shown to improve glucose homeostasis through an NEAA insufficiency-induced liver FGF21 axis [7]. FGF21 has also been shown to act directly on the brain to affect GnRH release [21,42] and influence ovarian follicle development [23]. We observed dose-dependent responses of hepatic Fgf21 expression and serum FGF21 concentration in rats fed with different dietary protein regimens, which prompted us to generate mice with liver-specific loss of FGF21 to investigate its role in the regulation of estrous cyclicity.

One of the most important findings is that the loss of hepatic FGF21 delayed the onset of disrupted estrous cyclicity when mice were fed the P4 diet, revealing that FGF21 plays a role in the regulation of fertility. This is consistent with a previous observation in which overexpression of hepatic FGF21 resulted in infertility caused by the downregulation of *Kiss*-1 expression in the hypothalamus [21]. In our study, the mRNA expression of *Fgfr1* in the hypothalamus of WT mice was increased when mice were fed the P4 diet. However, this effect was absent in FGF21LKO mice. Meanwhile, the FGF21LKO mice fed the P4 diet had higher hypothalamic *Kiss-1* expression than WT mice, suggesting that loss of hepatic FGF21 can enhance hypothalamic *Kiss-1* expression and promote estrous cyclicity. However, we cannot rule out the possibility that other factors are also involved. Additionally, we found that serum IGF1 concentration and hypothalamic Igf-1 receptor expression were higher in FGF21LKO mice fed the P4 diet. A previous study revealed that FGF21 can inhibit liver growth hormone signaling to decrease IGF-1 secretion [43], which may explain why FGF21LKO mice had a higher IGF-1 level than WT mice fed the P4 diet.

IGF-1 signaling has been reported to play a role in the maintenance of the normal estrous cycle, E_2_-induced preovulatory LH surge, and follicular development [44]. IGF-1 from the liver rather than the hypothalamus passes through the blood–brain barrier, binds to brain IGF-1R, and fluctuates during the estrous cycle, with the highest level at proestrus [45]. Interestingly, this is consistent with the gonadotropin-releasing effects of *Kiss*-1 neurons in the preovulatory phase [46]. Central or systemic administration of IGF-1 to immature rats results in an increase in *Kiss*-1 gene expression in the hypothalamus before pubertal onset, whereas an IGF-IR antagonist can block the IGF-1-induced increase in *Kiss*-1 gene expression in the nucleus [47]. Therefore, we conclude that IGF-1 was involved in the effects of dietary protein or FGF21 on the regulation of estrous cyclicity.

A certain amount of body fat is necessary for achieving and maintaining normal reproductive function [48]. The adipocyte hormone leptin, which controls appetite and body fat accumulation, links fat production to HPG axis activity [20]. Leptin replacement normalizes menstrual cycles, improves E_2_ levels, and stimulates gonadotropin secretion in women with lipodystrophy [48]. In the present study, rats fed protein-restricted diets had progressively increased body fat content, consistent with the results of a previous study [34]. The increased body fat content of these rats may be due to insufficient protein intake, in which energy is transformed into dietary fat rather than lean tissue. Additionally, protein restriction may induce hormonal changes to ensure higher efficiency for energy deposition, and this hypothesis was confirmed by the catch-up growth of the P4 rats that were re-fed the normal diet. Given the importance of adipose tissue in the regulation of reproductive function, if rats fed protein-restricted diets secrete more leptin based on increased body fat deposition, the protein-restricted rats should, at least partially, show a restoration of reproductive function. However, the increased body fat content in the protein-restricted rats did not associate with a higher circulating leptin concentration, inconsistent with the results of a previous study [34] in which serum leptin concentrations were greater in rats fed 5% and 8% casein diets than in control rats fed a 20% casein diet. This discrepancy may be explained by the fact that leptin secretion under protein restriction is dependent on physiological status. However, this hypothesis needs further investigation.

Interrupted female estrous cycles are associated with altered circulating amino acid levels. A previous study has reported that the lack of essential amino acids (EAA) in rats induces the cessation of the estrous cycle [49], whereas rats consuming a diet rich in EAA can rescue anestrus caused by caloric restriction [45]. A deficiency in dietary protein caused a decrease in EAA and an increase in non-essential amino acids (NEAA) in serum can influence brain function [50]. Interestingly, we found that the liver weight in rats was not significantly decreased, except in rats that were fed a diet with a protein content lower than 8%.

The liver, as the primary nutrient-sensing organ, integrates metabolic and reproductive functions via IGF-1 [45] and FGF21 [21,23], which is indicative of its role in the partition of nutrition between metabolic processes and reproductive function. When the activity of the HPG axis was suppressed by decreased protein intake, the lower E_2_ concentration inhibited hypothalamic kisspeptin expression as well as the activity of the HPG axis [51]. On the other hand, nutrition can be partitioned to increase the deposition of adipose tissue, and this finding may explain why P4 rats had a high percentage of body fat.

In the present study, the rats and mice fed the P4 diet had decreased bodyweight; therefore, disrupted estrous cyclicity and ovarian follicular development might be due to the decreased bodyweight, since bodyweight was a critical factor regulating estrous cyclicity [45]. Thus, one limitation in the present study is that we did not compare estrous cyclicity and ovarian follicular development using a bodyweight-paired control. In the mouse study, both the WT and FGF21LKO mice fed the P4 diet had similar bodyweight; however, the FGF21LKO mice fed the P4 diet showed delayed disruption of estrous cyclicity and downstream regulation, which indicated that FGF21 acted as a hormone mediating the effects of dietary protein restriction on the fertility, at least partly.

## 5. Conclusions

In conclusion, our results demonstrate that dietary protein restriction favors adipose tissue deposition at the expense of reproductive function. A diet consisting of 4% protein can disturb the estrous cycle, whereas disrupted estrous cyclicity can be restored by applying 18% dietary protein. FGF21 signaling in the liver and kisspeptin signaling in the hypothalamus are likely involved in this process. Based on these findings, obese woman should consume sufficient protein prior to becoming pregnant, especially if they are on a low-protein vegetarian diet or a low-protein diet to lose bodyweight.

## Figures and Tables

**Table 1:** Tissue weight of rats fed various levels of dietary protein.

Parameters	P4	P8	P13	P18	P23	SEM
BW (g)	249.24 ^b^	269.98 ^a^	277.16 ^a^	279.53 ^a^	279.26 ^a^	3.19
Ovary (g)	0.035 ^c^	0.045 ^b^	0.050 ^b^	0.058 ^a^	0.061 ^a^	0.003
Ovary (% BW)	0.014 ^d^	0.017 ^cd^	0.018 ^bc^	0.021 ^ab^	0.022 ^a^	0.001
Uterus (g)	0.50 ^c^	0.61 ^b^	0.65 ^ab^	0.73 ^a^	0.64 ^ab^	0.04
Uterus (% BW)	0.20 ^b^	0.23 ^ab^	0.24 ^ab^	0.26 ^a^	0.23 ^ab^	0.01
Fat pads (g)	13.28 ^ab^	13.88 ^a^	13.39 ^ab^	12.83 ^ab^	10.99 ^b^	0.90
Fat pads (% BW)	5.31 ^a^	5.14 ^a^	4.81 ^ab^	4.56 ^ab^	3.92 ^b^	0.31
Liver (g)	6.74 ^b^	7.41 ^a^	7.95 ^a^	7.75 ^a^	8.00 ^a^	0.21
Liver (% BW)	2.71	2.74	2.88	2.77	2.87	0.08

BW, bodyweight; P4, P8, P13, P18, and P23, dietary protein contents at 4%, 8%, 13%, 18%, and 23%.Means within a row followed by a different letter ^a,b,c,d^ are significantly different. *n* = 18, 18, 16, 13, and 18 rats for P4, P8, P13, P18, and P23 groups.

**Table 2:** Serum free amino acid concentrations of rats fed various levels of dietary protein.

Parameters	P4	P8	P13	P18	P23	SEM
Essential, μmol/L						
Threonine	280.31 ^b^	478.83 ^a^	481.81 ^a^	427.99 ^a^	524.74 ^a^	26.80
Valine	162.61 ^b^	219.32 ^a^	214.76 ^a^	227.42 ^a^	221.84 ^a^	10.38
Methionine	47.20	47.70	45.18	46.29	54.75	3.71
Isoleucine	100.74	125.03	113.89	136.30	131.39	12.82
Leucine	154.75 ^b^	230.03 ^a^	227.18 ^a^	277.60 ^a^	252.56 ^a^	21.32
Phenylalanine	88.97 ^c^	94.88 ^bc^	95.65 ^bc^	109.59 ^a^	107.88 ^ab^	3.41
Lysine	437.24 ^b^	440.75 ^b^	466.58 ^ab^	505.47 ^ab^	629.22 ^a^	53.14
Total	1271.81 ^c^	1636.54 ^b^	1645.05 ^ab^	1730.67 ^ab^	1922.38 ^a^	82.66
Nonessential, μmol/L						
Asparagine	56.58	60.46	67.59	66.36	62.77	6.25
Serine	741.73 ^a^	499.04 ^bc^	524.92 ^b^	380.37 ^d^	414.87 ^cd^	22.44
Glutamate	291.07 ^a^	277.28 ^a^	218.28 ^b^	218.16 ^b^	209.49 ^b^	13.95
Glycine	842.13 ^a^	419.05 ^b^	310.20 ^c^	312.43 ^c^	305.64 ^c^	18.80
Alanine	1047.00 ^a^	807.59 ^b^	491.24 ^c^	600.53 ^c^	615.79 ^c^	36.01
Citrulline	127.93 ^a^	91.57 ^b^	86.20 ^b^	89.39 ^b^	76.50 ^b^	6.55
Tyrosine	43.96 ^c^	55.96 ^bc^	66.41 ^b^	70.48 ^b^	95.88 ^a^	4.75
Ornithine	139.27	116.99	127.92	97.78	97.62	16.26
Histidine	68.67	69.57	58.04	64.99	62.59	4.82
Arginine	335.45	241.22	254.91	265.83	273.32	31.49
Proline	439.64 ^a^	256.90 ^b^	143.13 ^c^	149.44 ^c^	118.44 ^c^	17.03
Total	4133.43 ^a^	2895.62 ^b^	2348.83 ^c^	2315.75 ^c^	2332.90 ^c^	87.76
EAA/NEAA	0.31 ^d^	0.56 ^c^	0.70 ^b^	0.75 ^ab^	0.83 ^a^	0.03

BW, bodyweight; P4, P8, P13, P18, and P23, dietary protein contents at 4%, 8%, 13%, 18%, and 23%. Different letters in the same row indicate a significant difference at *p* < 0.05. *n* = 6 rats per group.

## Data Availability

The data in this study are available on reasonable request from the corresponding author.

## Supplementary Materials

The following supporting information can be downloaded at: https://www.mdpi.com/article/10.3390/nu15133049/s1. Table S1. Primer sequence for targeted genes. Table S2. Tissue development of rats after the re-feeding experiment ^1,2,3^. Table S3. Serum amino acid concentrations of rats after re-feeding experiment ^1,2^. Figure S1. Oestrus cyclicity of rats fed varied amount of protein. P4, P8, P13, P18, and P23 denote dietary protein content at the level of 4%, 8%, 13%, 18%, and 23%. P, proestrus; E, estrus; M, metestrus; D, diestrus.

## Abbreviations

FGF21	factor fibroblast growth factor 21;
HPG	hypothalamic–pituitary–gonadal
GnRH	gonadotropin-releasing hormone
E2	estradiol
GPR54	G-protein-coupled receptor 54
LH	luteinizing hormone
IGF-1	insulin-like growth factor-1
FGF21LKO	FGF21 liver-specific knockout
WT	wild-type
ELISA	enzyme-linked immunosorbent assay
EAA	essential amino acid
NEAA	non-essential amino acid
