# Supplementary material for: Dietary Protein Regulates Female Estrous Cyclicity Partially via Fibroblast Growth Factor 21
Source: Nutrients. 2023 Jul 6;15(13):3049. doi: 10.3390/nu15133049 (PMC10347210; doi:10.3390/nu15133049)
Supplement: Supplementary file 1 [file nutrients-15-03049-s001.zip › nutrients-2485807-supplementary.pdf]

nutrients

MDPI

Supplementary tables

Table S1. Primer sequence for targeted genes.

	Gene	Primer (5'-3')
Rat	*KiSS-1*	F: GCTGCTGCTTCTCCTCTGTGT<br>R: CTGTTGGCCTGTGGGTTCA
	Gpr-54	F: GGAACTCACTGGTCATCTTCGT<br>R: GTACGCAGCACAGAAGGAAAGT
	Esr-1	F: GGCTGCGCAAGTGTTACGAA<br>R: CATTTCGGCCTTCCAAGTCAT
	Fshr	F: GAATGATGTCTTGGAAGTAATAG<br>R: CTTAATGCCTGTGTTGGA
	LHcgr	F: AACAATGCGAAAGCACAGTTAGA<br>R: GCACATTGGAGT GTCTTGGGT
	Fgf21	F: CCTGGAGCTCAAAGCCTTGA<br>R: AAACTGCAGGCCTCAGGATC
	β-actin	F: CAACCGTGAAAAGATGACCCAG<br>R: ATGGGCACAGTGTGGGTGAC
Mouse	*KiSS-1*	F: CTCTGTGTCGCCACCTATGG<br>R: TTCCCAGGCATTAACGAGTTC
	Gpr-54	F: CCGTCCAACGCTTCAGGAT<br>R: GTGTAGCGAAAAACAGGGGAA
	Esr-1	F: CCCGCCTTCTACAGGTCTAAT<br>R: CTTTCTCGTTACTGCTGGACAG
	Fshr	F: TCTGGGCCACTCGTTTTACAC<br>R: TTGCATTCCAGTTGCATGGC
	LHcgr	F: ACGAGACGCTTTATTCTGCCA<br>R: AGGGGTACTTTGAAGGCAGC
	Fgf21	F: CTGGGGGTCTACCAAGCATA<br>R: CACCCA GGATTTGAATGACC
	Klb	F: CAACCCACTCCCATCTCGG<br>R: AGCACAGCTCA- GCGTAGTCC
	Fgfr1	F: AGAGTCCAAGAGTAAAAGCAGC<br>R: CTTCCGAGGTTCAGCTCTCC
	Cyp19a1	F: CGGGCTACGTGGATGTGTT<br>R: GAGCTTGCCAGGCGTTAAAG
	β-actin	F: GGCTGTATTCCCCTCCATCG<br>R: CCAGTTGGTAACAATGCCATGT

Gpr54, G protein-coupled receptor 54; *IGF-1R*, insulin-like growth factor-1 receptor; *Esr-1*, estrogen receptor α; *Fshr*, follicle stimulating hormone receptor; *LHcgr, luteinizing hormone/chorionic gonadotropin receptor; Fgf21, fibroblast growth factor 21; Klb, βKlotho; Fgfr1, fibroblast growth factor receptor 1;*. F, forward; R, reverse.

Table S2. Tissue development of rats after the re-feeding experiment [1,2,3].

	Bodyweight	Ovary		Uterus		Fat pads		Liver	
	(g)	(g)	(%)	(g)	(%)	(g)	(%)	(g)	(%)
P4+P18	281.80	0.041	0.015	0.79	0.28	14.73	5.20	8.51	3.02
P18+P18	287.96	0.051	0.018	0.80	0.27	14.98	5.18	8.59	2.98
SEM	8.3	0.004	0.001	0.06	0.02	1.13	0.27	0.31	0.06

[1] P4+P18 denotes P4 rats with disturbed estrous cyclicity were re-fed by P18 diets. P18+P18 denotes P18 rats with normal estrous cyclicity were continued on their diets. [2] Values are expressed as means with standard errors of means, n= 5 or 6 rats per group. [3] % denotes tissue weight to body weight ratio expressed as percentage.

Table S3. Serum amino acid concentrations of rats after re-feeding experiment [1,2].

Amino Acid	P18+P18	P4+P18	SEM
Essential, µmol/L			
Threonine	478.98	481.17	12.31
Valine	210.41	228.46	7.83
Methionine	46.65	45.13	2.92
Isoleucine	99.78	94.03	3.51
Leucine	219.27	228.38	7.94
Phenylalanine	71.37	67.66	4.17
Lysine	612.97	529.08	52.98
**Total**	1739.43	1673.91	61.30
Nonessential, µmol/L			
Asparagine	38.47	43.63	1.79
Serine	302.07	309.92	18.01
Glutamate	248.44	272.94	20.92
Glycine	238.32	236.44	15.14
Alanine	609.25	691.50	56.05
Citrulline	82.96	81.11	5.60
Tyrosine	70.81	85.92	6.67
Ornithine	59.80	60.81	6.07
Histidine	52.25	52.37	2.13
Arginine	172.86	161.42	12.48
Proline	238.37	234.04	14.30
Total	2113.59	2165.84	97.16
EAA/NEAA	0.83	0.78	0.03

[1] P4+P18 denotes P4 rats with disturbed estrous cyclicity were re-fed by P18 diets. P18+P18 denotes P18 rats with normal estrous cyclicity were continued on their diets. [2] Values are expressed as means with standard errors of means, n= 5 or 6 rats per group.

Supplemental Figures

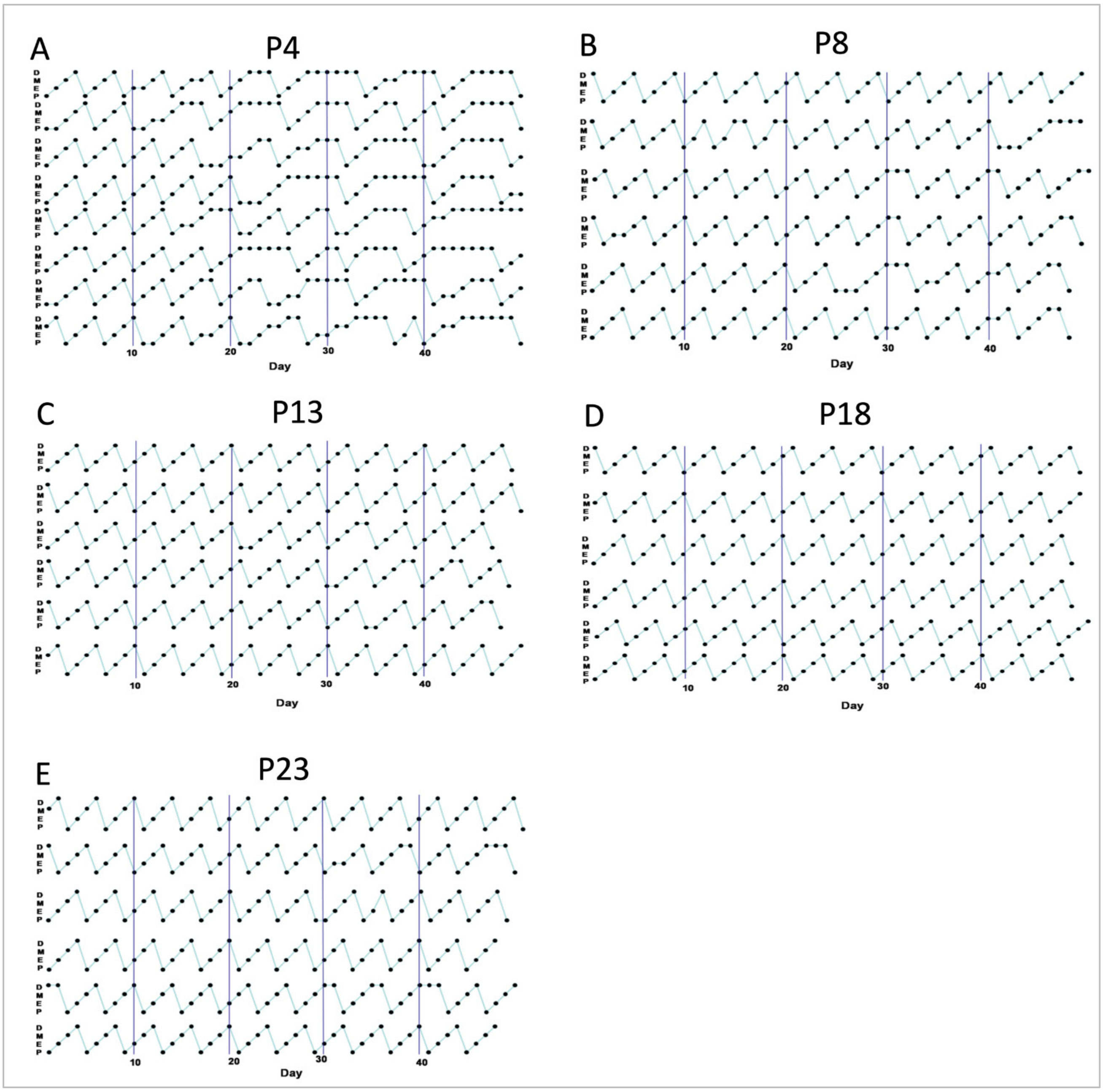

Figure S1. Oestrus cyclicity of rats fed varied amount of protein. P4, P8, P13, P18, and P23 denote dietary protein content at the level of 4%, 8%, 13%, 18%, and 23%. P, proestrus; E, estrus; M, metestrus; D, diestrus.
